# Association between prognostic factors and the clinical deterioration of preterm neonates with necrotizing enterocolitis

**DOI:** 10.1038/s41598-022-17846-0

**Published:** 2022-08-17

**Authors:** Ibnu Sina Ibrohim, Henggar Allest Pratama, Aditya Rifqi Fauzi, Kristy Iskandar, Nunik Agustriani, Gunadi

**Affiliations:** 1 Pediatric Surgery Division, Department of Surgery, Faculty of Medicine, Public Health and Nursing, Universitas Gadjah Mada/Dr, Sardjito Hospital, Yogyakarta, 55281 Indonesia; 2 Department of Child Health, Faculty of Medicine, Public Health and Nursing, Universitas Gadjah Mada, UGM Academic Hospital, Yogyakarta, 55291 Indonesia

**Keywords:** Medical research, Risk factors

## Abstract

Necrotizing enterocolitis (NEC) is responsible for most morbidity and mortality in neonates. Early recognition of the clinical deterioration in newborns with NEC is essential to enhance the referral and management and potentially improve the outcomes. Here, we aimed to identify the prognostic factors and associate them with the clinical deterioration of preterm neonates with NEC. We analyzed the medical records of neonates with NEC admitted to our hospital from 2016 to 2021. We ascertained 214 neonates with NEC. The area under the receiver operating characteristic (ROC) curve and cut-off level of age at onset, C-reactive protein (CRP), leukocyte count, and platelet count for the clinical deterioration of preterm neonates with NEC was 0.644 and 10.5 days old, 0.694 and 4.5 mg/L, 0.513 and 12,200/mm^3^, and 0.418 and 79,500/mm^3^, respectively. Late-onset, history of blood transfusion, thrombocytopenia, and elevated CRP were significantly associated with the clinical deterioration of neonates with NEC (*p* =  < 0.001, 0.017, 0.001, and < 0.001, respectively), while leukocytosis, gestational age, and birth weight were not (*p* = 0.073, 0.274, and 0.637, respectively). Multivariate analysis revealed that late-onset and elevated CRP were strongly associated with the clinical deterioration of neonates with NEC, with an odds ratio of 3.25 (95% CI = 1.49–7.09; *p* = 0.003) and 3.53 (95% CI = 1.57–7.95; *p* = 0.002), respectively. We reveal that late-onset and elevated CRP are the independent prognostic factor for the clinical deterioration of preterm neonates with NEC. Our findings suggest that we should closely monitor preterm neonates with NEC, particularly those with late-onset of the disease and those with an elevated CRP, to prevent further clinical deterioration and intervene earlier if necessary.

## Introduction

Necrotizing enterocolitis (NEC) is a severe gastrointestinal emergency that affects preterm neonates^1,2^. NEC is responsible for most perioperative fatalities in pediatric surgery, with a mortality rate of up to 19%^3^. However, studies from developing countries on the clinical deterioration in preterm neonates are minimal.

In addition, validated early indicators of clinical deterioration in preterm neonates with NEC are essential. Early predictors for surgery in premature neonates with NEC would help enhance referral and treatment pathways and potentially improve outcomes^4^. Here, we aimed to identify the prognostic factors and associate them with the clinical deterioration of preterm neonates with NEC.

## Methods

### Patients

A retrospective study was conducted using medical records of neonates with NEC at our institution from January 2016 to June 2021. We included 237 diagnosed premature neonates with NEC, with the International Classification of Diagnosis (ICD) X code of P.77. Subsequently, we excluded 10 and 13 neonates due to term neonates and incomplete medical records, respectively. We investigated 214 neonates for final analysis^5^.

### Staging of NEC

According to modified Bell's staging, the diagnosis and staging of NEC were established, consisting of the severity of systemic, intestinal, radiographic, and laboratory findings^6^.

### Prognostic factors and ROC curve

We evaluated the following prognostic factors for the clinical deterioration of preterm neonates with NEC: age at onset, C-reactive protein (CRP), leukocyte count, platelet count, history of packed red cell (PRC) transfusion, gestational age, and birth weight. The CRP level was determined using an immunochemical assay (NycoCard™ CRP, Abbott, US) with the normal value of < 5 mg/L. We defined clinical outcomes of NEC into two categories: worsened and improved. The clinical deterioration was defined as worsening of the modified Bell's staging. According to gestational age, the preterm birth was classified into the following: extremely preterm (< 28 weeks), very preterm (28 to < 32 weeks), and moderate to late preterm (32 to < 37 weeks)^7^. The birth weight was defined as normal birth weight (NBW) (≥ 2500 g), low birth weight (LBW) (< 2500 g), very low birth weight (VLBW) (< 1500 g), and extremely low birth weight (ELBW) (< 1000 g)^8^.

The cut-off value of age at onset, CRP, leukocyte count, and platelet count was analyzed by receiver operating characteristics (ROC) curves. According to a previous study, we assumed the cut-off level for late-onset NEC in preterm infants was ≥ 14 days^9^.

### Statistical analysis

The Chi-square test was used to determine the association between the prognostic factors and the clinical deterioration of premature neonates with NEC. The multivariate regression test was used to look for the strong prognostic factors for clinical deterioration of premature neonates with NEC. The *p*-value of < 0.05 was determined as a significant level. The IBM SPSS Statistics version 16 (SPSS Chicago, USA) was used for all statistical analyses.

### Ethics approval and consent to participate

This study was approved by the Institutional Review Board of the Faculty of Medicine, Universitas Gadjah Mada/Dr. Sardjito Hospital, Yogyakarta, Indonesia (KE/FK/0464/EC/2021). Written informed consent was obtained from all parents for participating in this study. The research has been performed following the Declaration of Helsinki.

## Results

### Baseline characteristics

A total of 214 preterm neonates with NEC were included in the study, with 77% improved clinical outcomes (Table 1).

**Table 1:** Baseline characteristics of neonates with NEC in our institution.

Characteristics	*N* (%)
Sex	
Male	98 (45.8)
Female	116 (54.2)
**History of PRC transfusion**	
Yes	112 (52.3)
No	102 (47.7)
**Clinical outcomes**	
Worsened	49 (23)
Improved	165 (77)

*NEC* necrotizing enterocolitis, *PRC* packed red cell.

s.

### Association between prognostic factors and clinical deterioration of preterm neonates with NEC

**Figure 1:**
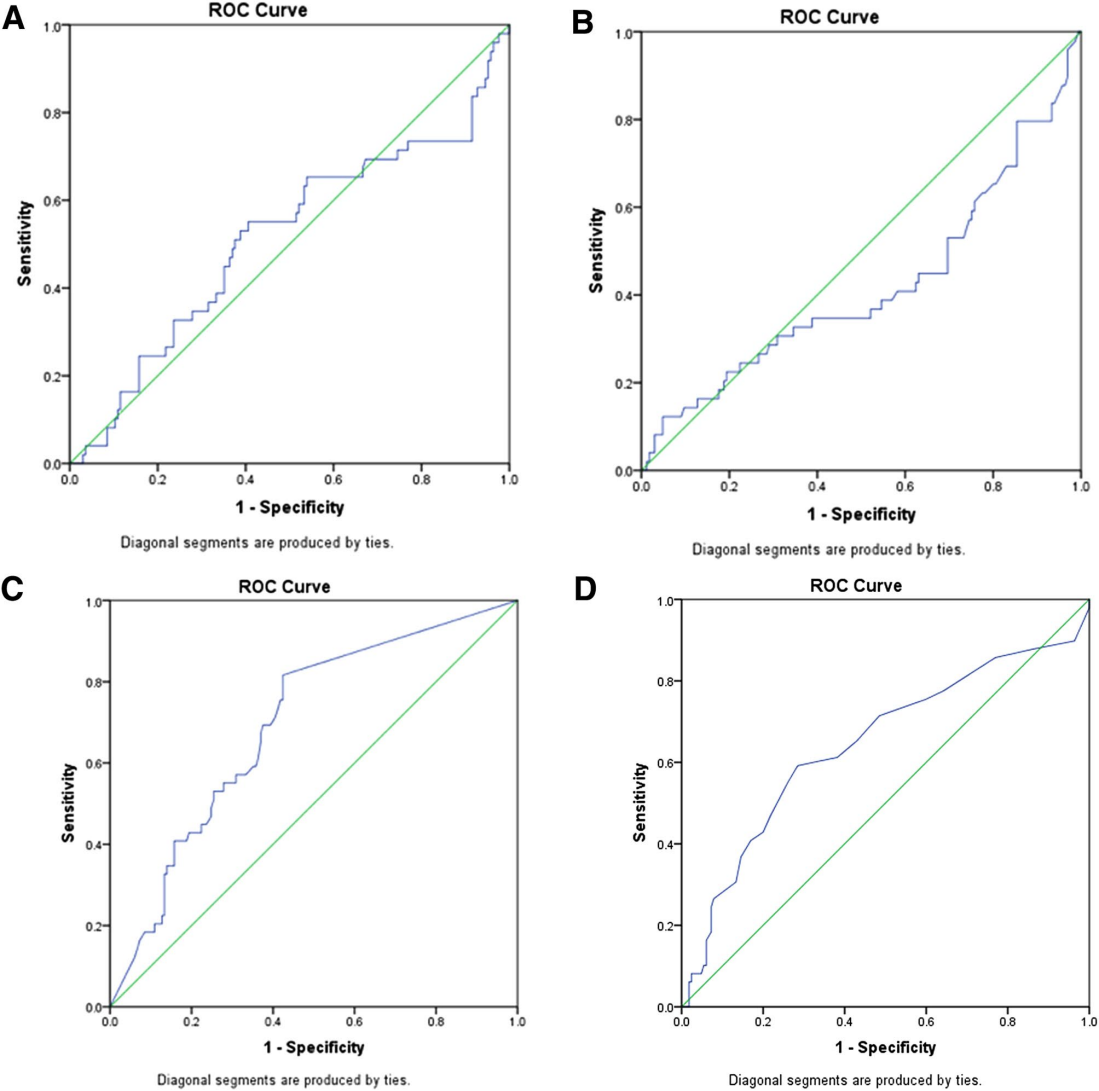
ROC curve of leucocyte count (**A**), platelet count (**B**), C-reactive protein (**C**), and the onset of NEC (**D**) for distinguishing between worsened and improved clinical deterioration of NEC, with the area under the ROC curve of 0.513, 0.418, 0.694, and 0.644, respectively.

The area under the receiver operating characteristic (ROC) curve and cut-off level of age at onset, C-reactive protein (CRP), leukocyte count, and platelet count for the clinical deterioration of preterm neonates with NEC was 0.644 and 10.5 days old, 0.694 and 4.5 mg/L, 0.513 and 12,200/mm3, and 0.418 and 79,500/mm3, respectively (Fig. 1).

Late-onset, history of blood transfusion, thrombocytopenia, and elevated CRP were significantly associated with the clinical deterioration of neonates with NEC (*p* =  < 0.001, 0.017, 0.001, and < 0.001, respectively), while leukocytosis, gestational age, and birth weight were not (*p* = 0.073, 0.274, and 0.637, respectively). (Table 2).

**Table 2:** Association between prognostic factors and clinical deterioration with NEC in our institution.

Variables	Clinical outcomes		OR (95% CI)	*p*-value
	Worsened (*N*, %)	Improved (*N*, %)		
**Age at onset (days)**				< 0.001*
≥ 10.5	29 (38.2)	47 (61.8)	2.63 (1.6–4.32)	
< 10.5	20 (14.5)	118 (85.5)		
**History of PRC transfusion**				0.017*
Yes	33 (29.5)	79 (70.5)	2.25 (1.15–4.39)	
No	16 (15.8)	86 (84.2)		
**Leukocyte count (/mm3)**				0.073
≥ 12,200	27 (28.7)	67 (71.3)	1.57 (0.96–2.57)	
< 12,200	22 (18.3)	98 (81.7)		
**Platelet count (/mm3)**				0.001*
≤ 79,500	27 (35.1)	50 (64.9)	2.18 (1.34–3.56)	
> 79,500	22 (16.1)	115 (83.9)		
**CRP (mg/L)**				< 0.001*
≥ 4.5	40 (36.4)	70 (63.6)	4.2 (2.15–8.23)	
< 4.5	9 (8.7)	95 (91.3)		
**Gestational age**				0.274
Extremely preterm	1 (7.1)	13 (92.9)	0.22 (0.03–1.78)	
Very preterm	16 (21.3)	59 (78.3)	0.79 (0.39–1.56)	
Moderate to late preterm	32 (25.6)	93 (74.4)		
**Birth weight**				0.637
ELBW	12 (30)	28 (70)	2.14 (0.41–11.29)	
VLBW	15 (20.3)	59 (79.7)	1.27 (0.25–6.43)	
LBW	20 (22.7)	68 (77.3)	1.47 (0.29–7.27_	
NBW	2 (16.7)	10 (83.3)		

*, *p* < 0.05; CI, confidence interval, *OR* odds ratio, *NEC* necrotizing enterocolitis, *PRC* packed red cell, *CRP* C-reactive protein, *NBW* normal birth weight, *LBW* low birth weight, *VLBW* very low birth weight, *ELBW* extremely low birth weight.

### Multivariate analysis of prognostic factors for clinical deterioration of preterm neonates with NEC

Multivariate analysis revealed that late-onset and elevated CRP were strongly associated with the clinical deterioration of neonates with NEC, with an odds ratio of 3.25 (95% CI = 1.49–7.09; *p* = 0.003) and 3.53 (95% CI = 1.57–7.95; *p* = 0.002), respectively (Table 3).

**Table 3:** Multivariate analysis of the clinical deterioration of premature neonates with NEC in our institution.

Variables	OR (95% CI)	*p*-value
Late-onset	3.25 (1.49–7.09)	0.003*
History of PRC transfusion	2.04 (0.95–4.39)	0.07
Thrombocytopenia	1.27 (0.59–2.75)	0.539
Elevated CRP	3.53 (1.57–7.95)	0.002*

*, *p* < 0.05; *CI* confidence interval, *OR* odds ratio, *NEC* necrotizing enterocolitis, *PRC* packed red cell, *CRP* C-reactive protein.

## Discussion

Since the survival of preterm newborns is continuously enhanced, it is advised that clinicians search for the modifiable prognostic factors for NEC to intervene earlier and prevent further clinical deterioration^10,11^. The frequency of clinical deterioration of our patients was 23%. It is similar to a previous study^12^. In addition, our study shows that late-onset and elevated CRP were the strong prognostic factors for the clinical deterioration of NEC with the OR of ~ 4 and fivefold, respectively. It is similar to a previous study^13^. CRP is an acute-phase reactant that rises in the bloodstream in response to infection or tissue damage. The liver generates it in response to infection or tissue damage that causes inflammation. CRP was shown to be higher in 83% of neonates with confirmed NEC at the time of diagnosis compared to those without NEC in a previous study^14^. Pourcyrous et al*.*^15^ recently used serial CRP measures to identify preterm infants with NEC in a large cohort. CRP levels were abnormal in both stages II and III of NEC^15^. They concluded that persistently high CRP levels in neonates with NEC might indicate persisting illness and/or complications and suggested serial CRP tests for NEC follow-up^15^. Notably, we do not have any data on blood culture. Therefore, our findings should be interpreted cautiously; mainly, the CRP might reflect the intestinal necrosis that occurs in NEC rather than infection.

In this study, the cut-off level of age at onset was 10.5 days. Our findings were compatible with another study^16^. We showed that premature neonates with late-onset NEC are fivefold more likely to deteriorate clinically than those with early-onset NEC. Our findings are compatible with a previous study^17^. However, previous studies revealed that the early onset of NEC is an independent prognostic factor for NEC to be managed surgically^16,18,19^. The timing of NEC development in term and preterm infants has been studied extensively in a prior study^20^. Early-onset NEC individuals were more likely to require surgery^20^. A previous study suggested that a more widespread immaturity of the intestinal innate immune response might contribute to the observed increased inflammation in the juvenile gut and hence play a role in the onset of NEC^21^.

There was a strong inverse association between gestational age and birth weight with NEC mortality^22,23^. However, our study did not show any association between gestational age and birth weight with the clinical deterioration of NEC. These differences might be due to several factors, including broad discrepancies in patient populations, disease degree, coexisting illnesses, and severity classification among institutions^24^. Moreover, the following important variables might explain the NEC occurring in neonates with LBW, such as the immaturity of the gastrointestinal tract and its function, barrier function, regulation of circulation, and immune defense^25^.

Thrombocytopenia is frequently found in NEC neonates and is a poor prognostic variable when platelet counts drop quickly^26^. Lower platelet counts were linked to the development of surgical NEC^26^. On the other hand, low platelet count was not found to be a reliable predictor of surgical NEC^17^. Intestinal ischemia can result in platelet thrombi and platelet consumption at the local level^27^. Furthermore, these platelet thrombi may hasten the bowel's necrosis process. Our study did not support thrombocytopenia as a significant prognostic factor for clinical deterioration of NEC.

PRC transfusion has been linked to the development of NEC in premature infants^28–30^. However, our study did not reveal a significant association between the history of PRC transfusion and clinical deterioration of preterm neonates with NEC. Christensen et al*.*^29^ found that neonates who had surgery for NEC after PRC transfusion had a greater mortality rate than infants who had surgery for NEC without PRC transfusion. However, these differences were not statistically significant. In addition, PRC transfusion is linked to an increased risk of surgical intervention^31^. This might be caused by the donated PRCs that are exposed to significant stress during the collection, preservation, and storage^32^. Red blood cells go through structural and metabolic changes that might impair intestinal oxygen transport^33,34^. The production of cytokines and other pro-inflammatory substrates after infusion of these products might cause an inflammatory response^33^.

In our cohort, leukocytosis did not correlate with the deterioration of premature NEC neonates. In most cases, leukocytosis is thought to be a response to inflammatory processes. Various etiologies, including infection, inflammation, medications, and stress, may lead to a change of leucocyte counts in newborns^35^.

Our study has limitations, such as a retrospective design and a single-center report, indicating that more multicenter research is needed to validate our findings. These facts should be considered during the interpretation of our results.

## Conclusions

We reveal that late-onset and elevated CRP are the independent prognostic factor for the clinical deterioration of preterm neonates with NEC. Our findings suggest that we should closely monitor preterm neonates with NEC, particularly those with late-onset of the disease and those with an elevated CRP, to prevent further clinical deterioration and intervene earlier if necessary.

## Data Availability

All data generated or analyzed during this study are included in the submission. The raw data are available from the corresponding author on reasonable request.

## Abbreviations

PRC: Packed red cell
CRP: C-reactive protein
CI: Confidence interval
OR: Odds ratio
NEC: Necrotizing enterocolitis
